# Comprehensive Analysis of Pyroptosis-Related Genes and Tumor Microenvironment Infiltration Characterization in Breast Cancer

**DOI:** 10.3389/fimmu.2021.748221

**Published:** 2021-09-30

**Authors:** JianBin Wu, Yuanyuan Zhu, MingMin Luo, Lei Li

**Affiliations:** ^1^ Department of Breast, Fujian Maternity and Child Health Hospital, Affiliated Hospital of Fujian Medical University, Fuzhou, China; ^2^ The First School of Clinical Medicine (Dongzhimen Hospital) , Beijing University of Chinese Medicine, Beijing, China; ^3^ Reproductive Medicine Center, Fujian Maternity and Child Health Hospital, Affiliated Hospital of Fujian Medical University, Fuzhou, China; ^4^ Department of Pathology, University of Otago, Dunedin, New Zealand

**Keywords:** breast cancer, pyroptosis, tumor microenvironment, immunotherapy, immune checkpoint inhibitor (ICI)

## Abstract

**Background:**

Immunotherapy has emerged as a significant strategy to treat numerous tumors. The positive response to immunotherapy depends on the dynamic interaction between tumor cells and infiltrating lymphocytes in the tumor microenvironment (TME). Pyroptosis, inflammation-induced cell death, is intricately associated with several tumors. However, the relationship between pyroptosis and clinical prognosis, immune cell infiltration, and immunotherapy effect is unclear in breast cancer (BRCA).

**Methods:**

We comprehensively evaluated 33 pyroptosis-related genes and systematically assessed the relationship between pyroptosis and tumor progression, prognosis, and immune cell infiltration. The PyroptosisScore was used to quantify the pyroptosis pattern of a single tumor patient. We then assessed their values for predicting prognoses and therapeutic responses in BRCA.

**Results:**

Three different modes of PyroptosisClusters were determined. The characteristics of TME cell infiltration in these three PyroptosisClusters were highly consistent with three immunophenotypes of tumors, including immune-excluded, immune-inflamed, and immune-desert phenotypes. Comprehensive bioinformatics analysis revealed that patients with a low PyroptosisScore had higher immune checkpoint expression, higher immune checkpoint inhibitor (ICI) scores, increased immune microenvironment infiltration, and were more sensitive to immunotherapy than those with a high PyroptosisScore.

**Conclusions:**

Our findings revealed the crucial role of pyroptosis in maintaining the diversity and complexity of TME. Pyroptosis is closely related to tumor progression, tumor prognosis, and immunotherapy response. Evaluating the PyroptosisScore of a single tumor can assist in understanding the characteristics of TME infiltration and lead to the development of more effective immunotherapy strategies.

## Background

The incidence of breast cancer (BRCA), one of the most common cancers in women worldwide, has been increasing annually at a high rate (1–3). Although the mortality rate of BRCA has been drastically reduced recently due to the development of more effective and superior medical diagnostic and imaging techniques, the prognosis of patients with BRCA is still poor (4, 5). BRCA is a highly heterogeneous cancer with different pathological characteristics and molecular subtypes. The tumor microenvironment (TME) has been implicated in the occurrence and development of BRCA (6–8). Studies have shown infiltration of numerous inflammatory cells in BRCA; e.g., the density of CD8+ T cells is highly related to the immune escape of BRCA. Similarly, the infiltration of CD8+ T and CD4+ T cells is significantly related to the prognosis of BRCA (9–11). Immunotherapy, especially immune checkpoint blocking (ICB), has emerged as the latest therapeutic approach for a variety of cancers (12, 13). However, compared with other cancers, little work has been done toward the development of immunotherapy for BRCA.

Pyroptosis refers to the Gasdermin family-induced programmed cell death and is accompanied by inflammatory and immune responses (14, 15). A complex relationship exists between pyroptosis and cancer; pyroptosis can not only inhibit the occurrence and development of tumors but also act as a pro-inflammatory signal to create a microenvironment suitable for tumor cell growth. Pyroptosis is primarily triggered by the activation of inflammasomes, which are induced by the canonical caspase-1 inflammasome pathway and the non-canonical caspase-4/5/11 inflammasome pathway (16–19). Increasing evidence shows the pivotal role of pyroptosis in the TME although the underlying mechanism of pyroptosis in BRCA microenvironment progression and the immune response is still unclear.

We comprehensively evaluated the expression of pyroptosis-related genes and their effect on the progression, malignancy, prognosis, and immune response of BRCA. We used the Cancer Genome Atlas (TCGA) database and Gene Expression Omnibus (GEO) to determine three different pyroptosis patterns in BRCA and evaluated the clinical characteristics, prognostic value, and immune infiltration level of the resulting PyroptosisClusters. In addition, we defined a PyroptosisScore that effectively predicted the prognosis of patients with BCRA and immunotherapy response. We believe that these findings can assist in the development of effective immunotherapies for BRCA.

## Materials and Methods

### BRCA Data Source and Preprocessing

Gene expression profiles and clinical information were downloaded from TCGA (https://portal.gdc.cancer.gov/) and GEO (https://www.ncbi.nlm.nih.gov/geo/) databases. The TCGA–BRCA cohort contained 1,109 BRCA samples and 113 normal tissue samples, and the GSE42568 cohort contained 104 BRCA samples and 17 normal tissue samples. For TCGA–BRCA cohort, fragments per kilobase million (FPKM) values were transformed into transcripts per million (TPM). The “sva” package of R software was used to address the batch effect. Patients whose survival information was unavailable were excluded from the study. A total of 1,200 patients were included in the study. The patients’ clinical information is given in **Supplementary Table 1**.

### Unsupervised Cluster Analysis

The pyroptosis-related literature revealed 33 pyroptosis-related genes (**Supplementary Table 2**) (20–22). Unsupervised clustering analysis was applied to identify distinct pyroptosis patterns based on the expression of 33 pyroptosis-related genes and classify the patients (TCGA–BRCA cohort and GSE42568 cohort) for further analysis. We used the R package “ConsensuClusterPlus” to perform the above analysis and 1000 times repetitions for guaranteeing the stability of clustering. The optimal number of clusters was determined according to the consensus clustering algorithm.

### Gene Set Variation Analysis

To study the differences in biological processes responsible for the characteristic patterns of pyroptosis, the “GSVA” R package was used to perform gene set variation analysis (GSVA) (23, 24). The “clusterProfiler” package was used for functional annotation and the gene set file (c2.cp.kegg.v7.2.symbols.gmt) was obtained from the MSigDB database (https://www.gsea-msigdb.org) (25–27).

### Estimation of TME Cell Infiltration, ImmuneScore, StromalScore, and ESTIMATEScore

We used the single-sample gene-set enrichment analysis (ssGSEA) program to measure the relative abundance of each cell infiltration in the BRCA TME. The ImmuneScore, StromalScore, and ESTIMATEScore were calculated using the “ESTIMATE” package (28–31).

### Generation of PyroptosisScore

A scoring system was established to quantitatively evaluate pyroptosis in individual BRCA patients. The process of establishing the scoring system is as follows: The DEGs identified from different pyroptosisclusters were firstly normalized among all samples and the overlap genes were extracted. Differential analysis and Venn diagram showed that there are 8 common differential genes among the three PyroptosisCluster. Then, we performed univariate Cox regression analysis for each gene. These genes with a significant prognosis were extracted for the next step of the analysis. We performed principal component analysis (PCA) to calculate pyroptosis scores using the following formula:


PyroptosisScore=Σ(PC1i+PC2i)

where i is the expression of pyroptosis-related genes (32, 33).

### Collection of Immunotherapy File

Immunophenoscore (IPS) is a good predictor of CTLA-4 and PD-1 responsiveness and thus the response to immunotherapy. The immune checkpoint inhibitor (ICI) immunophenoscore file was downloaded from the Cancer Immunome Database (TCIA, https://tcia.at/home) (34, 35).

### Statistical Analysis

All statistical analyses were conducted using the R (R 4.1.0) software. The student’s *t*-test (unpaired, two-tailed) was used to evaluate the differences between the two independent groups. One-way analysis of variance (ANOVA) and Kruskal–Wallis test were used as parametric and non-parametric methods, respectively, for data from more than two groups. The best cut-off score between the two groups with high and low PyroptosisScore was derived using the surv-cutpoint function. We applied the “limma” R package to identify the differentially expressed genes (DEGs) (36, 37). The enrichment analysis of gene ontology (GO), functional annotation, and Kyoto Encyclopedia of Genes and Genomes (KEGG) were performed using the “clusterProfiler” package. R packages “survival” and “survminer” were used for survival analysis (38, 39). The mutation landscape in patients was shown using the waterfall function of the “maftools” package (40, 41). If not specified above, *P*  < 0.05 was considered significant.

## Results

### Genetic Variation and Expression of Pyroptosis-Related Genes in BRCA

**Figure 1:**
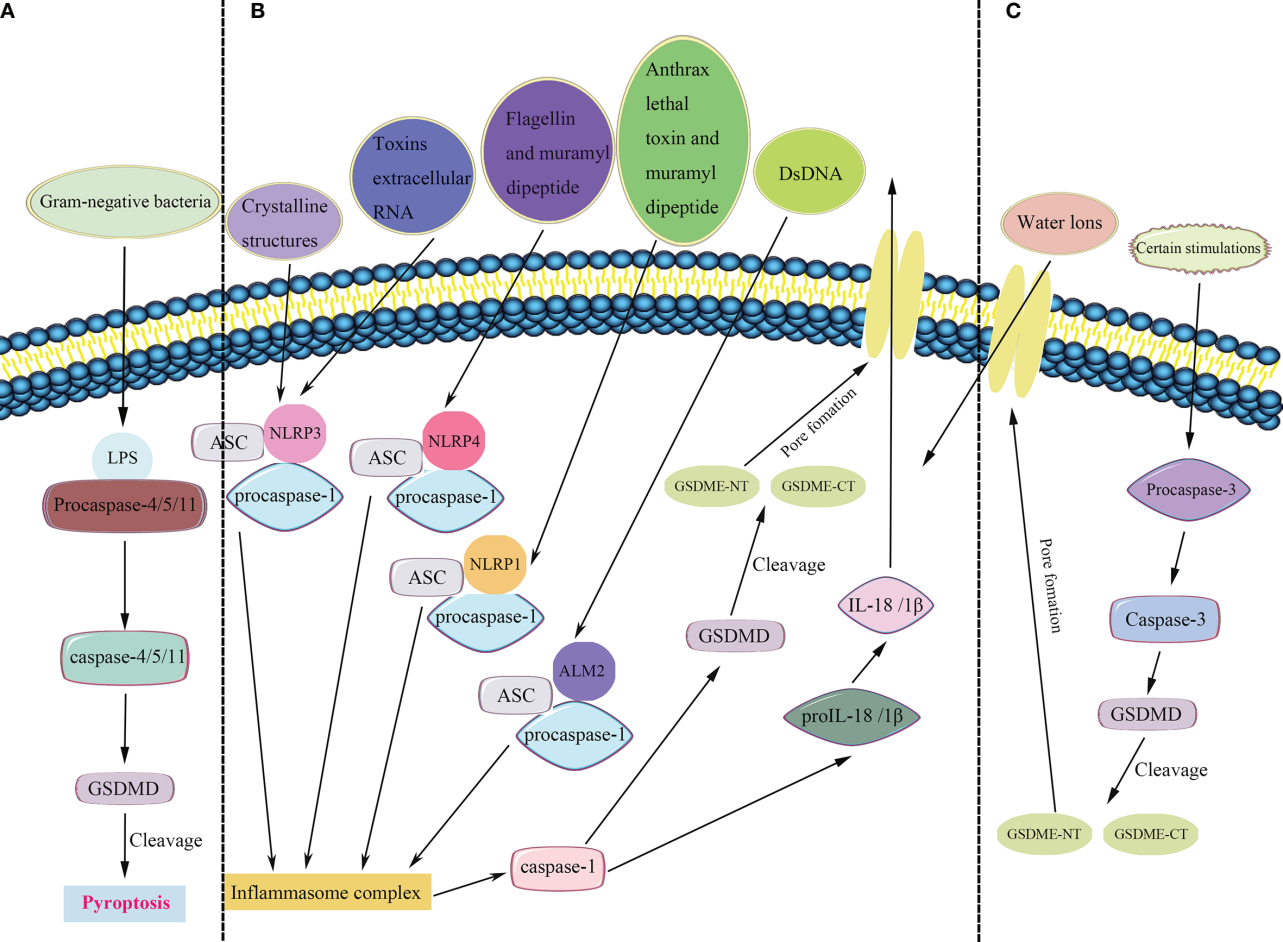
Pyroptosis pathways. **(A)** In the non-canonical inflammasome pathway, bacterial lipopolysaccharide (LPS) recognizes and activates caspase-4/5/11 to cleave gasdermin D (GSDMD) and induce pyroptosis. **(B)** In the canonical inflammasome pathway, the inflammasomes recruit and bind to ASC (apoptosis-associated speck-like protein containing a CARD), leading to ASC focus, which in turn recruits procaspase-1 and activates caspase-1. Caspase-1 is involved in the cleavage and maturation of proIL-18/1β and cleavage of GSDMD. The released N-terminal fragment of GSDMD (GSDMD-NT) creates pores in the plasma membrane, resulting in the secretion of IL-18/1β and causing water influx, and consequently cell swelling and osmotic lysis. The C-terminal fragment of GSDMD (GSDMD-CT) stays within the cytoplasm. **(C)** A new pyroptosis pathway revealed that certain stimulations activate caspase-3, a molecular considered as a substrate of apoptosis. Mature caspase-3 induces the cleavage of gasdermin E (GSDME), including its C-terminal and N-terminal fragments (GSDME-CT and GSDME-NT, respectively). Furthermore, GSDME-NT participants in the pore formation, resulting in pyroptosis.

**Figure 2:**
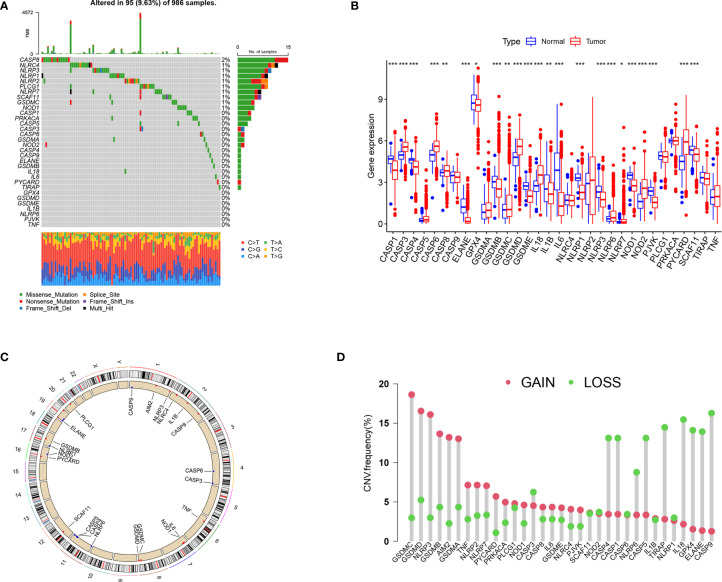
The landscape of genetic variation and expression of pyroptosis-related genes in BRCA. **(A)** Genetic alteration on a query of pyroptosis-related genes. **(B)** Gene expression levels of pyroptosis-related genes in BRCA compared to normal tissue. **(C)** The location of the CNV alteration of the pyroptosis-related genes changes on 23 chromosomes. **(D)** The frequency of CNV variation in pyroptosis-related genes. Red dots represent CNV amplification, while green dots represent CNV deletion. *P < 0.05, **P < 0.01, ***P < 0.001.

**Figure 1** summarizes the common pathways of pyroptosis. We analyzed the incidence of copy number variation and somatic mutations of pyroptosis-related genes in BRCA. Out of 986 samples, only 95 had mutations in pyroptosis-related genes, with a mutation frequency of 9.63% (**Figure 2A**). The mutation frequency of CASP8 was the highest (2%), and there were significant differences in the expression of certain genes (*ELANE*, *CASP5*, *CASP1*, *TNF*, *N0D2*, *IL18*, *NLRP7*, *NLRP3*, and *IL1B*) between CASP8 wild and CASP8 mutation groups. These results indicated that CASP8 has a mutational co-occurrence relationship with these genes (**Supplementary Figure 1**).

The expression analysis results showed that compared with the control group, the expression of *CASP3*, *CASP6*, *CASP8*, *GSDMD*, *IL18*, *NOD2*, and *PYCARD* was significantly higher in the tumor group, whereas that of *CASP1*, *CASP4*, *ELANE*, *GPX4*, *GSDMB*, *GSDMC*, *GSEMD*, *IL1B*, *IL6*, *NLRP1*, *NLRP3*, *NOD1*, *PJVK*, and *SCAF11* was significantly lower (**Figure 2B**). The results of survival analysis showed that the dysregulation of most pyroptosis-related genes was significantly related to prognosis (**Supplementary Figure 2**). The high expression of *CASP6*, *CASP5*, *TIRAP*, *SCAF11*, *NLRP7*, *PLCG1*, *GSDMC*, *GSDMD*, and *NLRC4* was associated with poor prognosis. The high expression of *ELANE*, *CASP9*, *CASP8*, *GSDMB*, *CASP4*, *CASP1*, *TNF*, *NOD1*, *PYCARD*, *NLRP6*, *NLRP3*, *NLRP2*, *IL6*, *NLRP1*, *IL18*, and *IL1B* was associated with a better prognosis.

The location of CNV (copy number variations) alteration of pyroptosis-related genes on chromosomes is shown in **Figure 2C**. The CNV alteration in pyroptosis-related genes was mostly related to amplification in the copy number, whereas *CASP3*, *CASP1*, *CASP4*, *NLRP6*, *CASP5*, *TIRAP*, *IL18*, *GPX4*, *ELANE*, and *CASP9* had a widespread frequency of CNV deletion (**Figure 2D**).

### Pyroptosis Patterns Mediated by 33 Pyroptosis-Related Genes in BRCA

**Figure 3:**
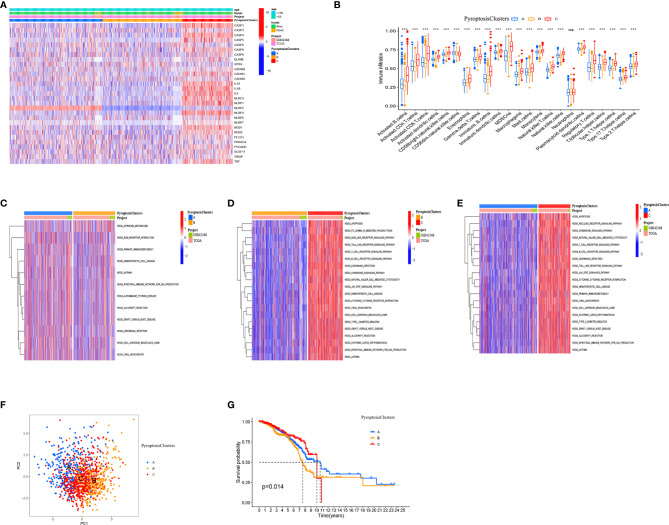
Pyroptosis patterns mediated by 33 pyroptosis-related genes in BRCA. **(A)** Heat map of the clinical relevance of three pyroptosis subtypes. **(B)** The abundance of each TME infiltrating cell in three PyroptosisCluster. **(C–E)** GSVA enrichment analysis showing the activation states of biological pathways in different pyroptosis modification patterns. The heat map was used to visualize these biological processes, and red represented activated pathways and blue represented inhibited pathways. **(F)** Principal component analysis (PCA) analysis of pyroptosis methylation modification pattern. **(G)** The overall survival of pyroptosis methylation modification pattern using Kaplan–Meier curves. *P < 0.05, **P < 0.01, ***P < 0.001; NS, Non Significance.

Our analysis cohort consisted of two BRCA datasets (TCGA–BRCA, GSE42568), OS data, and clinical information (**Supplementary Table 1**). A univariate Cox regression analysis used to screen pyroptosis-related genes associated with prognosis in BRCA showed that *IL18*, *CASP1*, *CASP4*, and *NLRP3* significantly correlated with prognosis (**Supplementary Table 3**). Based on the expression of pyroptosis-related genes, the “ConsensusClusterPlus” R package was used to classify patients with qualitatively different pyroptosis modification patterns. The three different modification patterns were determined using an unsupervised clustering analysis (**Supplementary Figure 3**) as PyroptosisCluster A, PyroptosisCluster B, and PyroptosisCluster C (**Figure 3A**).

We used the ssGSEA algorithm to analyze the immune cell infiltration of three clusters. We found that PyroptosisCluster C was remarkably rich in innate immune cell infiltration including B cells, CD4 T cells, CD8 T cells, macrophages, eosinophils, mast cells, MDSCs, mast cells, and T helper cells (**Figure 3B**). Moreover, we found that patients in the PyroptosisCluster C had a survival advantage (**Figure 3G**). The results of GSVA enrichment analysis showed that PyroptosisCluster A was significantly enriched in matrix pathways, such as ECM receptor interaction, cell adhesion molecules (CAMs) (**Figure 3C**). PyroptosisCluster C presented enrichment pathways associated with immune activation including the T cell receptor signaling pathway, B cell receptor signaling pathway, NOD-like receptor signaling pathway, Toll-like receptor signaling pathway, chemokine signaling pathway, cytokine receptor interactions, and JAK/STAT signaling pathway (**Figure 3E**), whereas PyroptosisCluster B was prominently related to immune suppression (**Figure 3D**). In addition, we found significant differences in the transcription profiles of pyroptosis-related genes between the three different PyroptosisClusters (**Figure 3F**). Based on the above analysis, we classified the PyroptosisCluster A as an immune rejection phenotype, characterized by innate immune cell infiltration and matrix activation. The PyroptosisCluster B was classified as an immune desert phenotype, characterized by immunosuppression. The PyroptosisCluster C was classified as an immunoinflammatory phenotype, characterized by adaptive immune cell infiltration and immune activation.

### Generation of Pyroptosis-Related Genes Signatures

**Figure 4:**
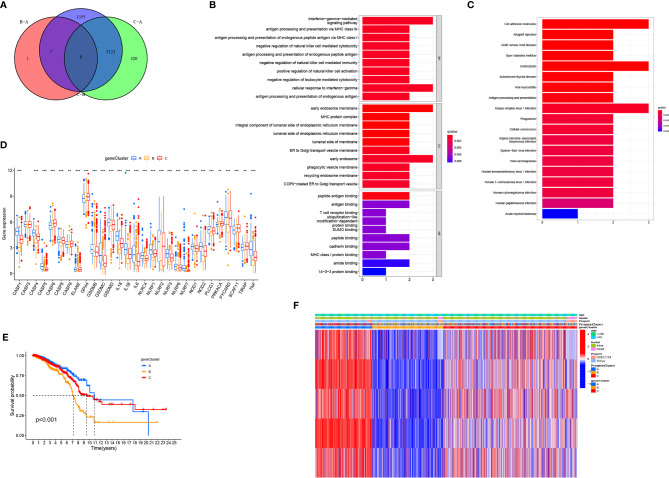
Generation of pyroptosis-related genes signatures. **(A)** Venn diagram showing overlapping genes of three PyroptosisCluster subtypes. **(B, C)** Results of GO and KEGG enrichment. **(D)** Gene expression levels of pyroptosis-related genes in three geneCluster. **(E)** Kaplan–Meier curves showing the overall survival of geneCluster. **(F)** Heat map of the clinical relevance of PyroptosisCluster and geneCluster. *P < 0.05, **P < 0.01, ***P < 0.001.

To further study the potential biological characteristics of pyroptosis-related genes, we identified eight overlapping genes, namely *NLRP2*, *GPR132*, *HLA-E*, *FXYD5*, *CDH3*, *HLA-F*, *PML*, and *MSN* in the three subtypes (PyroptosisCluster A, PyroptosisCluster B, and PyroptosisCluster C) and performed KEGG and GO enrichment analyses (**Figure 4A**). These genes showed enrichment of biological processes significantly related to pyroptosis and immunity, confirming that pyroptosis plays a crucial role in the immune regulation of TME (**Figures 4B, C**). To further verify this regulatory mechanism, we performed an unsupervised cluster analysis using the obtained genes. The results were consistent with the clustering grouping of PyroptosisCluster (**Supplementary Figure 4**). The unsupervised clustering algorithm revealed three different pyroptosis genome phenotypes, termed gene clusters A–C. We observed significant differences in the expression of pyroptosis-related genes in three gene clusters, which was consistent with the expected results (**Figure 4D**). The results of survival analysis showed that gene cluster A showed a significant survival advantage, and gene cluster B was significantly related to poor prognosis (**Figure 4E**). The heat map shows clinical characteristics of PyroptosisCluster and gene cluster (**Figure 4F**). The opposite characteristics were observed in gene cluster A and gene cluster B.

### Generation of PyroptosisScore

**Figure 5:**
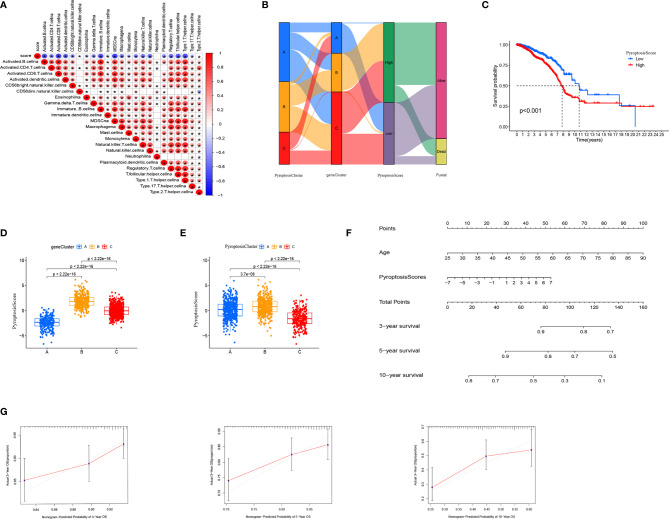
Generation of PyroptosisScore. **(A)** Correlation between PyroptosisScore and immune cell infiltration. **(B)** Alluvial diagram showing the connection between pyroptosis cluster, gene cluster, and pyroptosis score. **(C)** The overall survival of PyroptosisScore. **(D)** The level of PyroptosisScore in different genecluster sub-groups. **(E)** The level of PyroptosisScore in different PyroptosiscCluster subgroups. **(F)** Nomogram to predict 3-, 5- and 10-year OS in the TCGA cohort. **(G)** Calibration plots of the nomogram to predict OS at 3-, 5- and 10-year. *P < 0.05, **P < 0.01, ***P < 0.001.

To further analyze the functions of pyroptosis in BRCA, we constructed a scoring system, termed as PyroptosisScore, based on these pyroptosis genes to quantify the pyroptosis pattern of individual BRCA patients. We found that the majority of immune cells were negatively correlated with PyroptosisScore (**Figure 5A**). The alluvial diagram was used to visualize the changes in the attributes of individual patients (**Figure 5B**). To study the relationship between PyroptosisScore and patients’ prognosis, the “survminer” program was used to find the best cut-off value. The patients were divided into high and low PyroptosisScore groups. The high PyroptosisScore group was associated with a poor prognosis (**Figure 5C**). In addition, both gene cluster B (**Figure 5D**) and PyroptosisCluster B (**Figure 5E**) had a high PyroptosisScore, whereas both gene cluster B (**Figure 4E**) and PyroptosisCluster B (**Figure 3G**) had a poor prognosis. This finding also validated our analysis results. The results of the nomogram plot showed that PyroptosisScore may have a good advantage in long-term survival prediction (**Figure 5F**). The calibration chart showed that PyroptosisScore had a good performance, with a harmonious consistency (C index = 0.69) between the predicted and observed survival rates (**Figure 5G**).

### Clinical, Tumor Somatic Mutation, and Tumor Microenvironment Characteristics of PyroptosisScore in TCGA–BRCA Cohort

**Figure 6:**
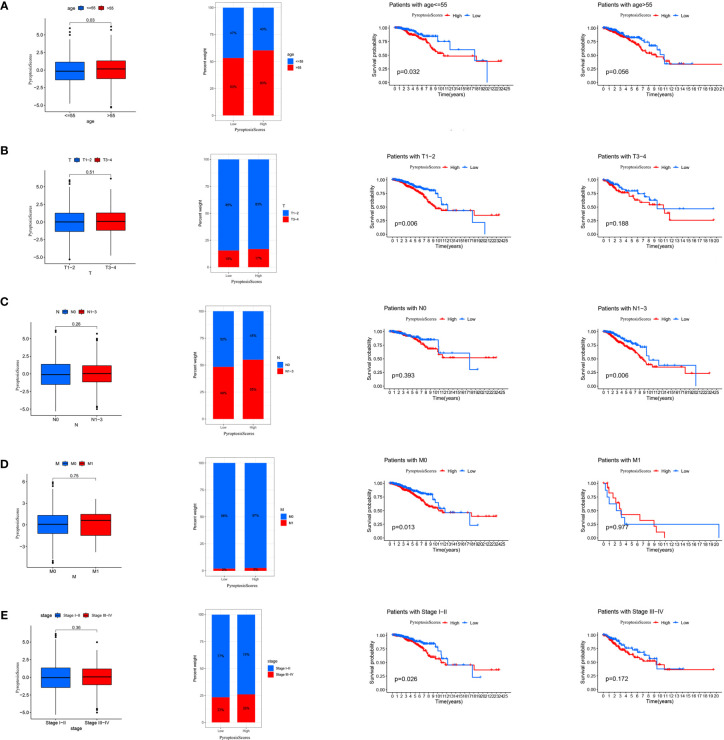
The relationship between PyroptosisScore and clinic in the TCGA-BRCA cohort. The relationship between age **(A)**, T **(B)**, M **(C)**, N **(D)**, stage **(E)** and PyroptosisScore. The TNM system of cancer staging reflects the extent of tumor growth, where primary tumor (T), nodal status for metastasis (N), and metastasis at the distant organs (M).

**Figure 7:**
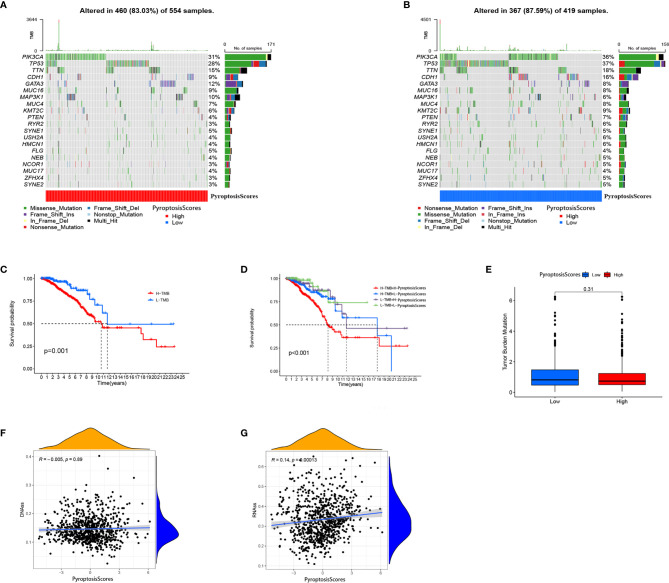
Characteristics of tumor somatic mutation in different PyroptosisScore groups. **(A)** Waterfall plot of tumor somatic mutation created by groups with a high PyroptosisScore. **(B)** The waterfall plot of tumor somatic mutation created by groups with a low PyroptosisScore. **(C)** Kaplan–Meier curves were used to perform survival analyses for patients with low and high TMB. **(D)** The overall survival of patients was stratified by both PyroptosisScore and TMB using Kaplan–Meier curves. **(E)** The TMB level was higher in the low PyroptosisScore group. There was no significant correlation between DNAss **(F)**, RNAss **(G)**, and PyroptosisScore.

The clinical correlation analysis revealed that patients older than 55 years had a higher PyroptosisScore (**Figure 6A**). In the patient subgroup, the high PyroptosisScore of types <55 age, T1-2, N1-3, M0, and stage I–II patients was significantly correlated with poor prognosis (**Figures 6A–E**). The R package “maftools” was used to visualize the differences in the distribution of somatic mutations in the high (**Figure 7A**) and low (**Figure 7B**) PyroptosisScore groups. We found that the mutation rate of the two was similar (83.03% and 87.59%). The results of survival analysis showed that the higher tumor mutation burden (TMB) had a poor prognosis (**Figure 7C**). Among these, H-TMB+H-PyroptosisScore had the worst prognosis (**Figure 7D**). In addition, no significant differences in TMB between the high and low PyroptosisScore groups were observed (**Figure 7E**). Furthermore, no significant correlation between PyroptosisScore and DNAss (**Figure 7F**) and RNAss (**Figure 7G**) was observed.

**Figure 8:**
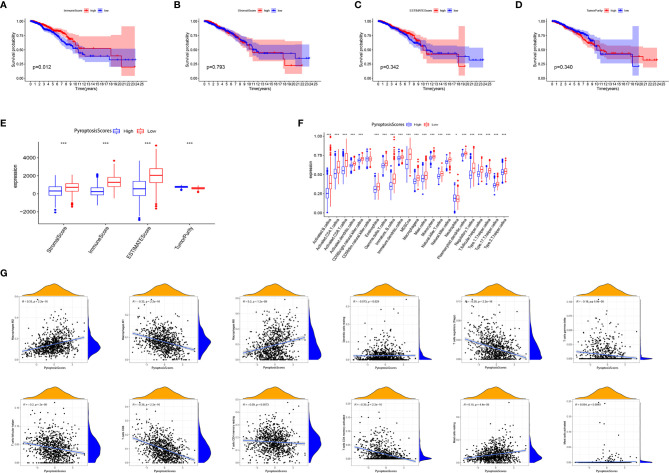
The role of PyroptosisScore in the tumor microenvironment. Survival analysis of ImmuneScore **(A)**, StromalScore **(B)**, ESTIMATEScore **(C)**, and TumorPurity **(D)** in BRCA patients. **(E)** The level of ImmuneScore, StromalScore, ESTIMATEScore, and TumorPurity was significantly different in the high and low PyroptosisScore subgroups. **(F)** The abundance of each TME-infiltrating cell in high and low PyroptosisScore subgroups. **(G)** Correlation analysis between PyroptosisScore and immune infiltrating cells. *P < 0.05, **P < 0.01, ***P < 0.001.

We further analyzed the characteristics of TME of patients with BRCA and found that a high ImmuneScore was significantly associated with a better prognosis (**Figure 8A**). No significant correlation between ImmuneScore (**Figure 8B**), ImmuneScore (**Figure 8C**), TumorPurity, (**Figure 8D**), and the prognosis was present. Except for TumorPurity, ImmuneScore, ImmuneScore, and ImmuneScore, all were significantly expressed in the low PyroptosisScore group (**Figure 8E**). In addition, we found that the low PyroptosisScore group had a more abundant immune cell infiltration pattern (**Figure 8F**). These findings emphasized the impact of pyroptosis on the BRCA microenvironment and validated our conclusion that the low PyroptosisScore group had more survival advantages than the high PyroptosisScore group (**Figure 5C**). The results of correlation analysis showed that PyroptosisScore was significantly and positively correlated with macrophages M2, macrophages M0, and resting mast cells. A significant negative correlation was observed with macrophages M1, regulatory T cells, gamma delta T cells, follicular helper T cells, CD8^+^ T cells, and CD4^+^ memory-activated T cells (**Figure 8G**).

### PyroptosisScore in Immunotherapy

**Figure 9:**
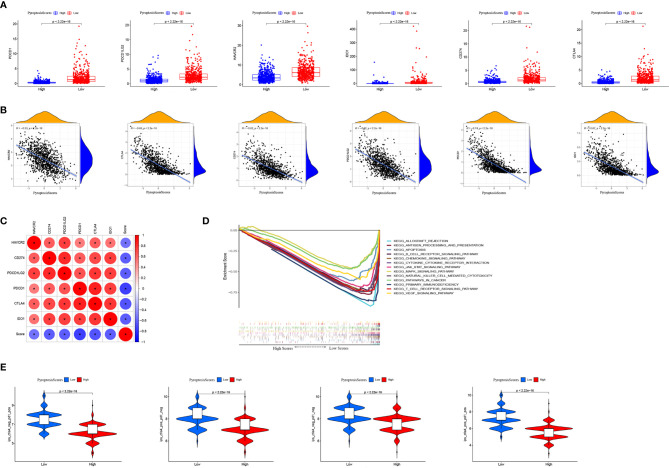
The role of PyroptosisScore in immunotherapy. **(A)** The expression of immune checkpoints in high and low PyroptosisScore subgroups. **(B)** Correlation analysis between PyroptosisScore and immune checkpoint. **(C)** Correlation analysis between immune checkpoints. **(D)** GSEA enrichment analysis between high and low PyroptosisScore subgroups. **(E)** Immunotherapy response between high and low PyroptosisScore subgroups.

Immunotherapy, especially immune checkpoint blocking therapy, has recently emerged as an important player in the treatment of tumors. We analyzed certain key immune checkpoint genes (*PDCD1*, *PDCD1LG2*, *HAVCR2*, *IDO1*, *CD274*, and *CTLA4*) in BRCA. The expression of these six immune checkpoint genes was significantly higher in the low PyroptosisScore group than in the high PyroptosisScore group (**Figure 9A**). In addition, PyroptosisScore had a significant negative correlation with these six immune checkpoint genes (**Figure 9B**). These six immune checkpoint genes were positively correlated with each other (**Figure 9C**). The results of GSEA enrichment analysis showed that the low PyroptosisScore group was enriched in cancer and immune-related pathways, such as antigen processing and presentation, apoptosis, B cell receptor signaling pathway, chemokine signaling pathway, cytokine receptor interactions, JAK/STAT signaling pathway, MAPK signaling pathway, natural killer cell-mediated cytotoxicity, cancer pathways, primary immunodeficiency, T cell receptor signaling pathway, and VEGF signaling pathway (**Figure 9D**). The ICI therapy represented by PD-L1 and PD-1 blockade is an effective method to treat certain tumors. To further evaluate the application of PyroptosisScore in BRCA, we obtained the immunotherapy profile of BRCA patients from the TCIA database and found that the low PyroptosisScore group had a higher ICI score and was more sensitive to immunotherapy than the high PyroptosisScore group (**Figure 9E**). In summary, tumors patients with low PyroptosisScore were characterized by inflammation, abundant immune infiltration, high expression of immune checkpoints, and a better prognosis and response to immunotherapy.

## Discussion

BRCA is the most common cancer among women globally, with a high morbidity and mortality rate. Numerous studies have emphasized the use of immune system cells as an effective treatment option for various cancers. However, the development of immunotherapy for BRCA is still in its initial stages, and only a few BRCA patients can benefit from immunotherapy. Therefore, there is an urgent need to determine new treatment strategies to improve the prognosis and treatment of BRCA.

Increasing evidence has implicated pyroptosis in the occurrence and development of tumors. Pyroptosis has been shown to inhibit tumor growth in skin cancer, colorectal cancer, and liver cancer (42–46). Pyroptosis can activate the innate immune system, inhibit the development of tumor cells by changing the TME, and even directly kill tumor cells. However, its functions in the BRCA microenvironment and immune response remain elusive.

In this study, we first explored the genetic variations and expression of pyroptosis-related genes based on the TCGA–BRCA and GSE42568 cohorts. Although the mutation frequency of pyrolysis-related genes was low, the majority of them were dysregulated in BRCA patients and related to prognosis. We next classified patients with BRCA according to the expression of pyroptosis-related genes. The unsupervised clustering algorithm resulted in three different pyroptosis patterns (PyroptosisCluster A, PyroptosisCluster B, and PyroptosisCluster C). Among these, PyroptosisCluster C was remarkably enriched in innate immune cell infiltration including B cells, CD4^+^ T cells, CD8^+^ T cells, macrophages, eosinophils, mast cells, MDSCs, and T helper cells. In addition, the cluster significantly corresponded to enrichment pathways associated with immune activation including the T cell receptor signaling pathway, B cell receptor signaling pathway, NOD-like receptor signaling pathway, Toll-like receptor signaling pathway, chemokine signaling pathway, cytokine receptor interaction, and JAK/STAT signaling pathway. We classified the PyroptosisCluster C as an immunoinflammatory phenotype, characterized by adaptive immune cell infiltration and immune activation, with a high survival advantage. Similar to the clustering results of PyroptosisCluster, three genomic subtypes were identified based on pyroptosis-related genes. These subtypes were also significantly related to immune activation, confirming the significant role of pyroptosis in immune regulation in TME landscapes.

The low PyroptosisScore group showed a more abundant immune cell infiltration pattern. The TME has been shown to contribute to the occurrence and development of BRCA (47, 48). The study by Bin-Zhi Qian et al. showed massive inflammatory cell infiltration in the TME of BCRA, with the infiltration of CD8^+^ and CD4^+^ T cells significantly related to the prognosis of BRCA (49–51). The density of CD8^+^ T cells is highly associated with immune escape in BRCA; we found that patients in the low PyroptosisScore group had a higher degree of CD8^+^ and CD4^+^ T cell infiltration, which was also in line with our expected results. The GSEA results showed that cancer and immune-related pathways were significantly enriched in the low PyroptosisScore group. Further research results showed that the low PyroptosisScore group had a higher level of immune checkpoint gene expression (*PDCD1*, *PDCD1LG2*, *HAVCR2*, *IDO1*, *CD274*, and *CTLA4*), a higher ICI score in CTLA-4/PD-1 immunotherapy, and was more sensitive to immunotherapy than the high PyroptosisScore group. We classified the patients in the low PyroptosisScore group as having hot tumors, characterized by abundant immune cell infiltration in TME and increased sensitivity to immunotherapy. Our findings confirmed the significant role of pyroptosis in shaping different substrates and immune TME landscapes, with a crucial impact on the therapeutic effect of immune checkpoint blockade. We believe that PyroptosisScore can be used as a prognostic biomarker for predicting patient survival, the efficacy of adjuvant chemotherapy, and the clinical response of patients to anti-CTLA-4/PD-1 immunotherapy. In addition, our study provides new insights for cancer immunotherapy, reidentifies “cold tumors” as “hot tumors”, and contributes to the development of new immunotherapies.

Although this study may have a good clinical guiding role, some limitations should be considered. Regarding the sample size, data from TCGA and GEO are not enough. More information needs to be collected. In addition, further experimental research and clinical research to verify our conclusions.

## Conclusion

In conclusion, pyroptosis plays a crucial role in the TME and prognosis of BRCA. Evaluating the PyroptosisScore of a single tumor can enhance our understanding of the characteristics of TME infiltration and assist in developing more effective immunotherapy strategies.

## Data Availability Statement

The data sets analyzed during the current study are available in the TCGA (https://portal.gdc.cancer.gov/), accession numbers TCGA-BRCA, BRCA-FPKM; GEO repository (https://www.ncbi.nlm.nih.gov/geo/query/acc.cgi?acc=GSE42568).

## Author Contributions

LL designed the study and wrote the manuscript. JW, YZ, and ML analyzed data and contributed to writing the manuscript. All authors contributed to the article and approved the submitted version.

## Conflict of Interest

The authors declare that the research was conducted in the absence of any commercial or financial relationships that could be construed as a potential conflict of interest.

## Publisher’s Note

All claims expressed in this article are solely those of the authors and do not necessarily represent those of their affiliated organizations, or those of the publisher, the editors and the reviewers. Any product that may be evaluated in this article, or claim that may be made by its manufacturer, is not guaranteed or endorsed by the publisher.

## Supplementary Material

Supplementary Figure 1 The expression levels of pyroptosis-related genes in CASP8 wild group and CASP8 mutant group.

Supplementary Figure 2 Prognostic analysis of pyroptosis-related genes in BRCA.

Supplementary Figure 3 Unsupervised clustering identifies three different Pyroptosis clusters. **(A)** Consensus clustering matrix for k = 3. **(B)** Consensus clustering cumulative distribution function (CDF) for k = 2–10. **(C)** Relative change in the area under CDF curve for k = 2–9. **(D)** Distribution of each sample in different clusters when k ranges from 2 to 9.

Supplementary Figure 4 Unsupervised clustering identifies three different geneCluster. **(A)** Consensus clustering matrix for k = 3. **(B)** Consensus clustering cumulative distribution function (CDF) for k = 2–10. **(C)** Relative change in the area under CDF curve for k = 2–9. **(D)** Distribution of each sample in different clusters when k ranges from 2 to 9.

## Abbreviations

BRCA, Breast cancer; TME, Tumor microenvironment; ICB, Immune Checkpoint Blocking; ICI, immune checkpoint inhibitor; FPKM, Fragments per Kilobase Million; TPM, Transcripts per Million; GO, Gene ontology; KEGG, Kyoto Encyclopedia of Genes and Genomes; DEG, Differential gene; CNV, copy number variations.
