# Supplementary figures and images for: Comprehensive Analysis of Pyroptosis-Related Genes and Tumor Microenvironment Infiltration Characterization in Breast Cancer
Source: Front Immunol. 2021 Sep 30;12:748221. doi: 10.3389/fimmu.2021.748221 (PMC8515898; doi:10.3389/fimmu.2021.748221)

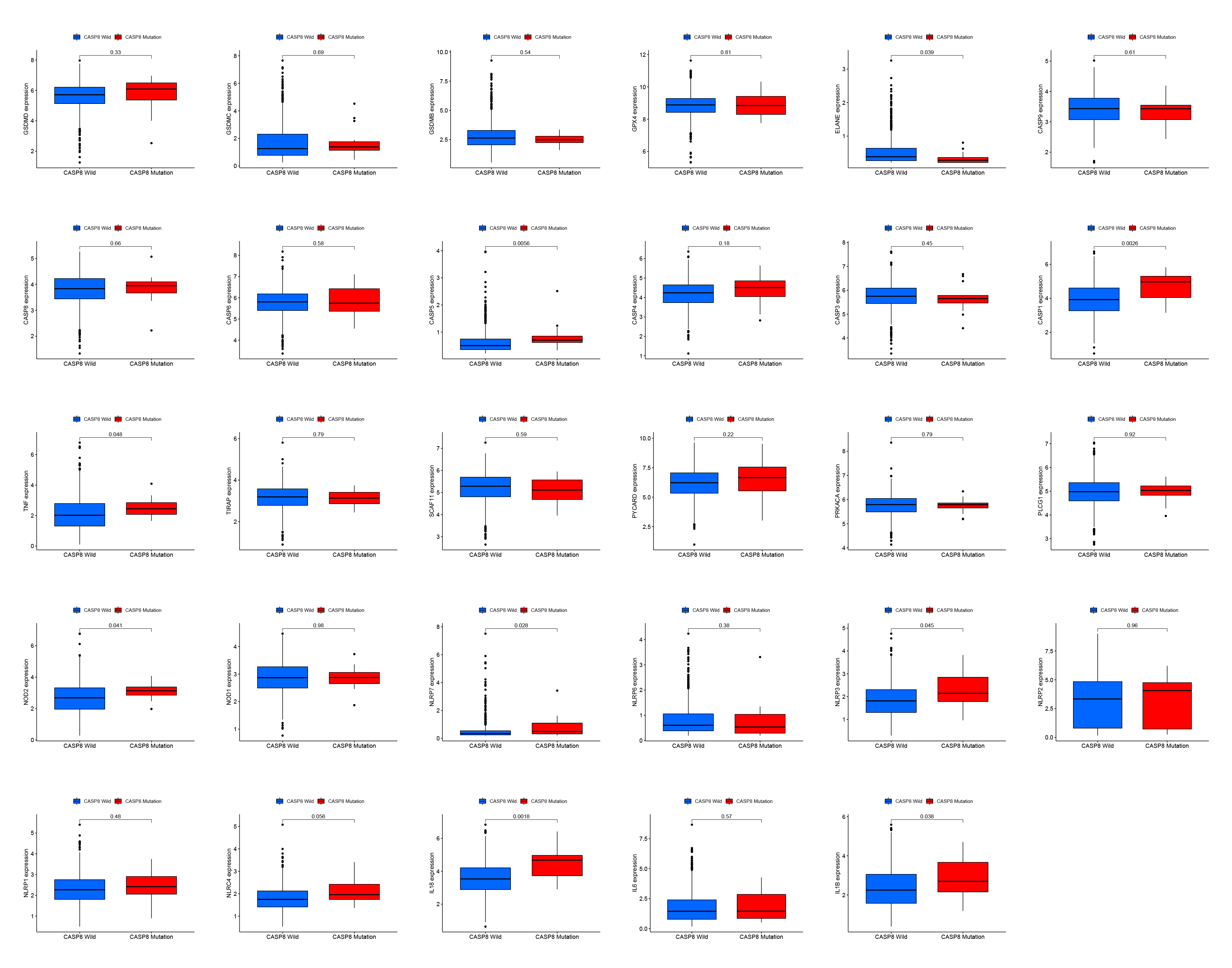

Supplement: Supplementary Figure 1 — The expression levels of pyroptosis-related genes in CASP8 wild group and CASP8 mutant group. [file Image_1.tif]

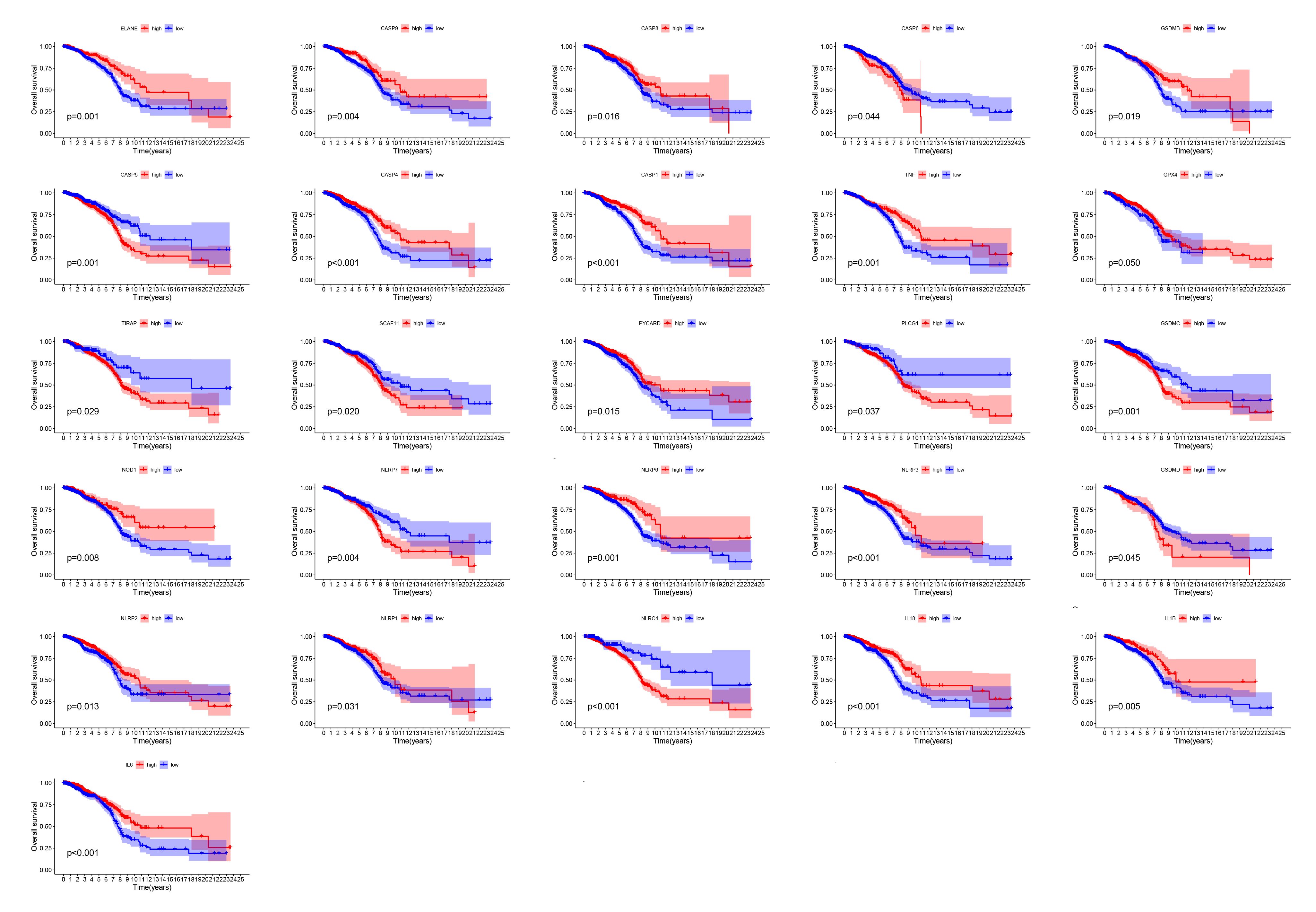

Supplement: Supplementary Figure 2 — Prognostic analysis of pyroptosis-related genes in BRCA. [file Image_2.tif]

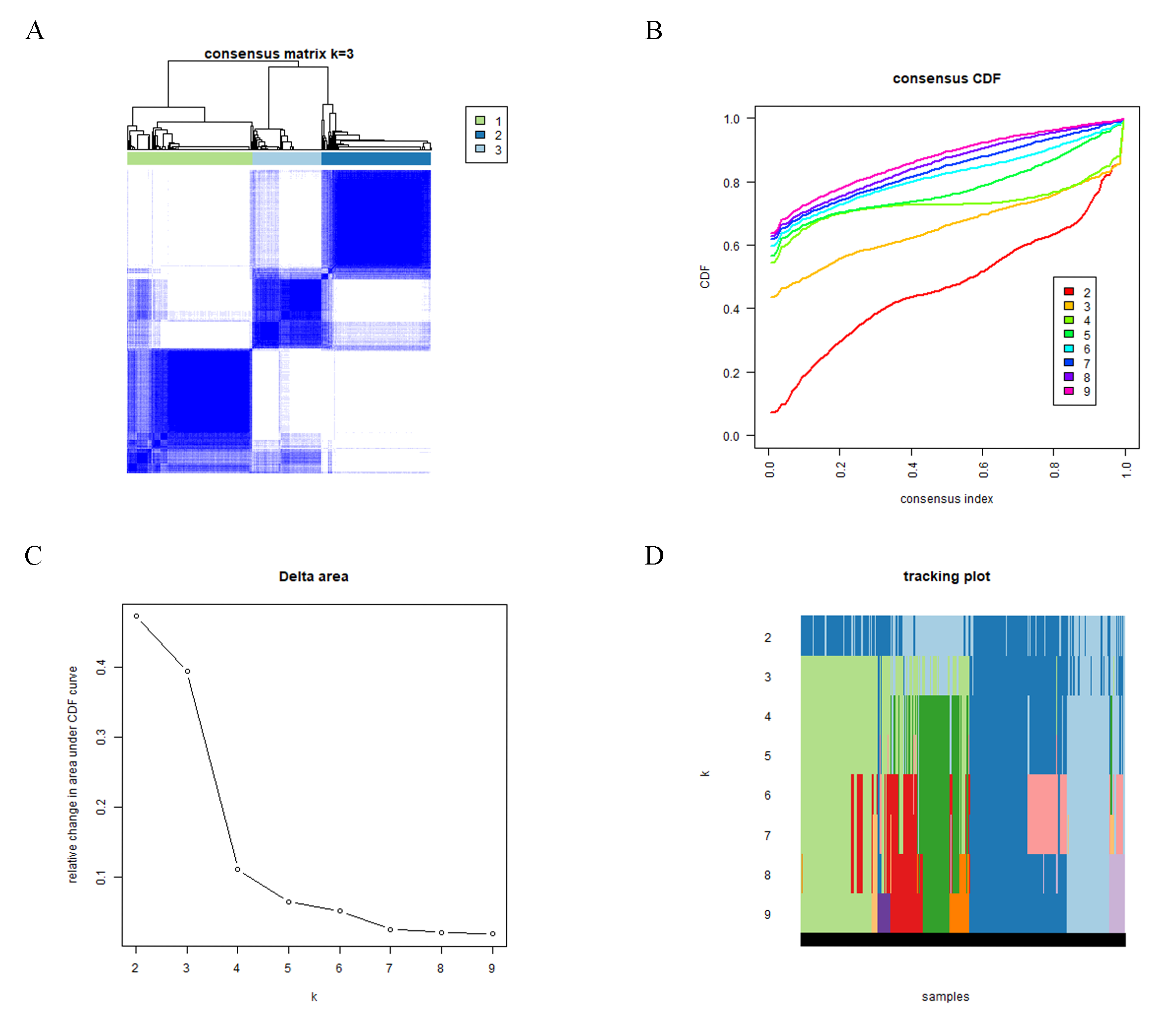

Supplement: Supplementary Figure 3 — Unsupervised clustering identifies three different Pyroptosis clusters. (A) Consensus clustering matrix for k = 3. (B) Consensus clustering cumulative distribution function (CDF) for k = 2–10. (C) Relative change in the area under CDF curve for k = 2–9. (D) Distribution of each sample in different clusters when k ranges from 2 to 9. [file Image_3.tif]

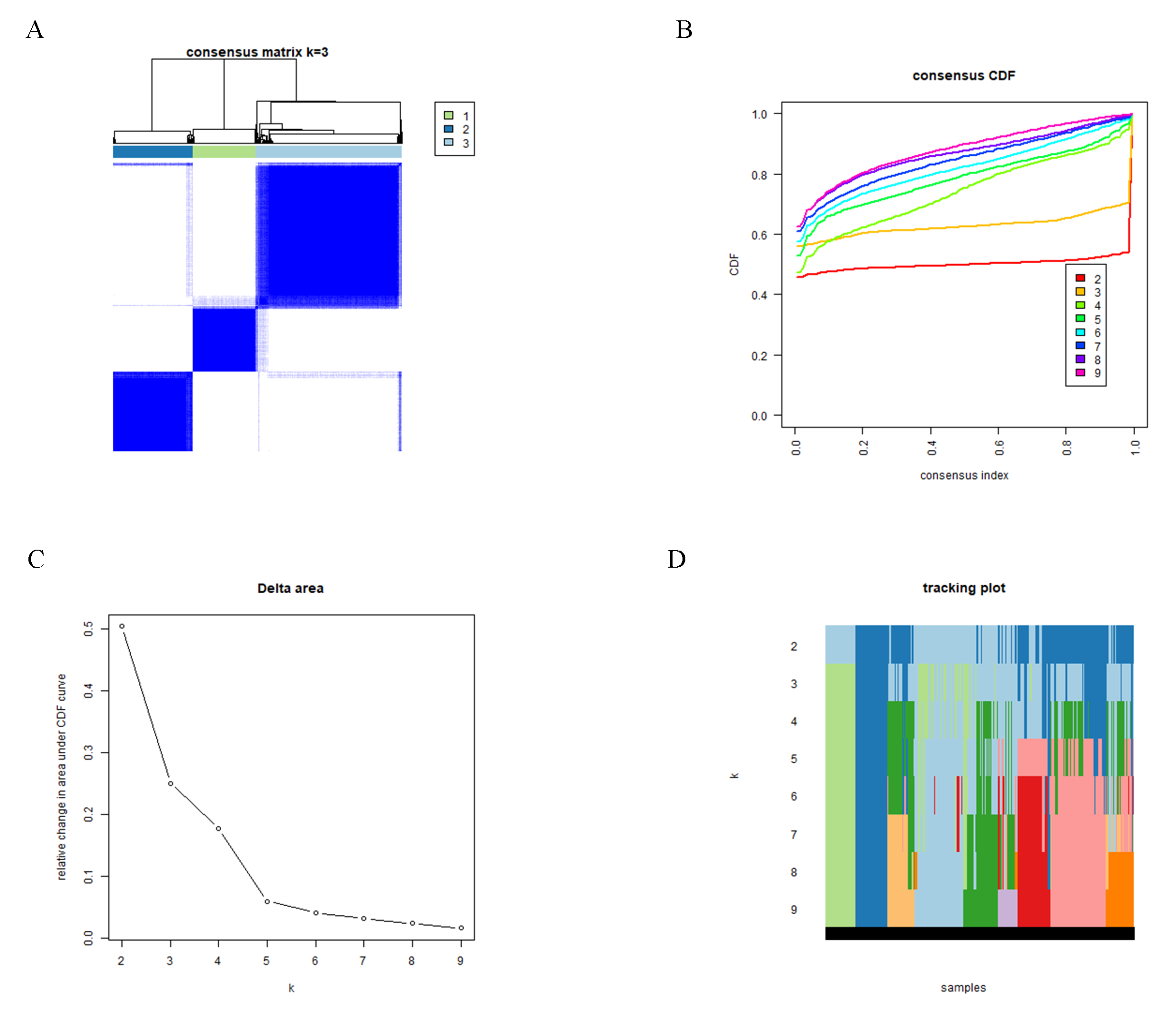

Supplement: Supplementary Figure 4 — Unsupervised clustering identifies three different geneCluster. (A) Consensus clustering matrix for k = 3. (B) Consensus clustering cumulative distribution function (CDF) for k = 2–10. (C) Relative change in the area under CDF curve for k = 2–9. (D) Distribution of each sample in different clusters when k ranges from 2 to 9. [file Image_4.tif]
