# Supplementary material for: Comprehensive Analysis of Pyroptosis-Related Genes and Tumor Microenvironment Infiltration Characterization in Breast Cancer
Source: Front Immunol. 2021 Sep 30;12:748221. doi: 10.3389/fimmu.2021.748221 (PMC8515898; doi:10.3389/fimmu.2021.748221)
Supplement: Supplementary file 5 [file Table_1.docx]

**TABLE. S1 Clinical Information.**

| Covariates | Type | TCGA-BRCA | GSE42568 |
| --- | --- | --- | --- |
| Age | <=55 | 471 (42.9%) | 46 (44.2%) |
| Age | >55 | 626 (57.1%) | 58 (55.8%) |
| Gender | female | 1085 (98.9%) | unknow |
| Gender | male | 12 (1.1%) | unknow |
| Stage | Stage I | 183 (16.7%) | unknow |
| Stage | Stage II | 621 (56.6 ) | unknow |
| Stage | Stage III | 249 (22.7%) | unknow |
| Stage | Stage IV | 20 (1.8%) | unknow |
| Stage | unknow | 24 (2.2%) | unknow |
| T | T1 | 281 (25.6%) | unknow |
| T | T2 | 635 (57.9%) | unknow |
| T | T3 | 138 (12.6%) | unknow |
| T | T4 | 40 (3.6%) | unknow |
| T | unknow | 3 (0.3%) | unknow |
| M | M0 | 912 (83.1%) | unknow |
| M | M1 | 22 (2.0%) | unknow |
| M | unknow | 163 (14.9%) | unknow |
| N | N0 | 516 (47.0%) | unknow |
| N | N1 | 364 (33.2%) | unknow |
| N | N2 | 120 (10.9%) | unknow |
| N | N3 | 77 (7.0%) | unknow |
| N | unknow | 20 (1.9%) | unknow |

**TABLE. S2 Pyroptosis-related genes.**

| Genes | Full-names |
| --- | --- |
| AIM2 | Absent in melanoma 2 |
| CASP1 | cysteine-aspartic acid protease-1 |
| CASP3 | cysteine-aspartic acid protease-3 |
| CASP4 | cysteine-aspartic acid protease-4 |
| CASP5 | cysteine-aspartic acid protease-5 |
| CASP6 | cysteine-aspartic acid protease-6 |
| CASP8 | cysteine-aspartic acid protease-8 |
| CASP9 | cysteine-aspartic acid protease-9 |
| ELANE | elastase, neutrophil expressed |
| GPX4 | glutathione peroxidase 4 |
| GSDMA | gasdermin A |
| GSDMB | gasdermin B |
| GSDMC | gasdermin C |
| GSDMD | gasdermin D |
| GSDME | gasdermin E |
| IL18 | interleukin 18 |
| IL1B | interleukin 1 beta |
| IL6 | interleukin 6 |
| NLRC4 | NLR family CARD domain containing 4 |
| NLRP1 | NLR family pyrin domain containing 1 |
| NLRP2 | NLR family pyrin domain containing 2 |
| NLRP3 | NLR family pyrin domain containing 3 |
| NLRP6 | NLR family pyrin domain containing 6 |
| NLRP7 | NLR family pyrin domain containing 7 |
| NOD1 | nucleotide binding oligomerization domain containing 1 |
| NOD2 | nucleotide binding oligomerization domain containing 2 |
| PJVK | pejvakin/deafness, autosomal recessive 59 |
| PLCG1 | phospholipase C gamma 1 |
| PRKACA | protein kinase cAMP-activated catalytic subunit alpha |
| PYCARD | PYD and CARD domain containing |
| SCAF11 | SR-related CTD associated factor 11 |
| TIRAP | TIR domain containing adaptor protein |
| TNF | tumor necrosis factor |

**TABLE. S3 Univariate Cox Result.**

| id | | HR | | HR.95L | | HR.95H | pvalue | | | km |
| --- | --- | --- | --- | --- | --- | --- | --- | --- | --- | --- |
| CASP1 | | 0.802554228903676 | | 0.701539086036425 | | 0.9181146184886 | 0.00135202303577874 | | | 8.3161798885012e-06 |
| CASP3 | | 1.04787749587555 | | 0.830616619634745 | | 1.32196638064535 | 0.693219821774111 | | | 0.247564157231699 |
| CASP4 | | 0.751542388524606 | | 0.628041501874051 | | 0.899329041255844 | 0.00181834044920042 | | | 4.3453795438686e-05 |
| CASP5 | | 0.868237848660296 | | 0.626967521303311 | | 1.20235408730458 | 0.395005521849644 | | | 0.00112228121070701 |
| CASP6 | | 1.07786661081945 | | 0.875285913165727 | | 1.32733363263832 | 0.480234786635467 | | | 0.0435846246849324 |
| CASP8 | | 0.827796550840696 | | 0.652853080454401 | | 1.049619202389 | 0.118718536425993 | | | 0.0164635351084094 |
| CASP9 | | 0.83755301325903 | | 0.623379449583974 | | 1.12530987424664 | 0.239409376986469 | | | 0.00399550162614914 |
| ELANE | | 0.761400046815515 | | 0.517081234479106 | | 1.12115851946295 | 0.167367327620207 | | | 0.00130905541260917 |
| GPX4 | | 0.881758336857657 | | 0.723949666778654 | | 1.07396660333805 | 0.211037923008805 | | | 0.0495971923426533 |
| GSDMB | | 0.972703101699682 | | 0.865924185612274 | | 1.09264914847849 | 0.640861521007048 | | | 0.0185490933464011 |
| GSDMC | | 1.05803605432149 | | 0.959686402917014 | | 1.16646467933857 | 0.257080001345217 | | | 0.00127302559590459 |
| GSDMD | | 0.99842744189264 | | 0.857318460045931 | | 1.16276203439137 | 0.983848917876288 | | | 0.0451245684538651 |
| IL18 | | 0.807307203712807 | | 0.7094171884711 | | 0.918704722352722 | 0.00117189390092245 | | | 2.17625337635141e-05 |
| IL1B | | 0.931242813284583 | | 0.816971606426286 | | 1.06149732802548 | 0.28620847340734 | | | 0.00505515520570554 |
| IL6 | | 0.898969851936898 | | 0.804055296379425 | | 1.00508857827372 | 0.0613716810952155 | | | 0.000523325137814612 |
| NLRC4 | | 1.01334401940646 | | 0.796863342664843 | | 1.28863513564676 | 0.913911762925685 | | | 0.000495587685005905 |
| NLRP1 | | 0.91328032570759 | | 0.757890565463269 | | 1.10052953728843 | 0.340441042800161 | | | 0.0306872485577937 |
| NLRP2 | | 0.959653653097508 | | 0.899097135570338 | | 1.02428880870497 | 0.215588949216062 | | | 0.0125880011180094 |
| NLRP3 | | 0.818872311265327 | | 0.671910156056273 | | 0.99797845904395 | 0.0477018454353487 | | | 9.96041820383597e-05 |
| NLRP6 | | 0.846315960989443 | | 0.65576917227885 | | 1.0922299127549 | 0.199805597980347 | | | 0.0010295888997498 |
| NLRP7 | | 0.828321710411752 | | 0.629612248913347 | | 1.08974508854239 | 0.178347195821981 | | | 0.00432642379525727 |
| NOD1 | | 0.926398234541949 | | 0.736215135288811 | | 1.16571046671809 | 0.514334886221646 | | | 0.00804377700704562 |
| NOD2 | | 1.00525918511662 | | 0.878873982848541 | | 1.14981902864621 | 0.93900753552982 | | | 0.0741144389873822 |
| PLCG1 | | 1.15895534911437 | | 0.928210279350877 | | 1.44706165307729 | 0.192813013776544 | | | 0.0367289174646961 |
| PRKACA | | 0.935007537181403 | | 0.667266826357298 | | 1.31017916679393 | 0.696232470288435 | | | 0.0994246363341534 |
| PYCARD | | 0.957917756676969 | | 0.861676114387796 | | 1.06490874382537 | 0.426125446744823 | | | 0.015249208793035 |
| SCAF11 | | 1.15162402312393 | | 0.942033671850227 | | 1.40784552640385 | 0.168400972001704 | | | 0.0201547302337475 |
| TIRAP | | 1.18631068367446 | | 0.954976752514681 | | 1.47368303416215 | 0.12265839320183 | | | 0.028839774417283 |
| TNF | 0.955019354238955 | | 0.849856938346083 | | 1.07319470585951 | | | 0.43940168678078 | 0.00102067574734277 | |
